# Acute pericarditis revealing COVID-19 infection: Case report

**DOI:** 10.1016/j.amsu.2021.01.053

**Published:** 2021-01-21

**Authors:** Raid Faraj, Chifaa Belkhayat, Amine Bouchlarhem, Ghizlane El Aidouni, Houssam Bkiyar, Brahim Housni

**Affiliations:** a Intensive Care Unit, Mohammed VI University Hospital, Faculty of Medicine and Pharmacy, Oujda, Morocco; b Radiology Department, Mohammed VI University Hospital, Faculty of Medicine and Pharmacy, Oujda, Morocco

**Keywords:** Acute pericarditis, Pericardial effusion, COVID 19, Colchicine

## Abstract

The COVID-19 is a global pandemic that is now responsible for more than 2 million deaths around the world. Its clinical manifestations are well known such as fever, fatigue and other respiratory signs like severe cough, dyspnea. Cardiac involvement, however, is less recognized and often underestimated and could be the only manifestation of COVID-19. Case presentation: We report a case of pericarditis as the primary presentation of COVID-19 among a young, healthy individual with no medical background, in the absence of the conventional respiratory signs. The diagnosis was based on a set of clinical, biological, radiological and electrocardiographic findings. In this case, the treatment was based on the use of Colchicine in addition to COVID-19 treatment. The outcome was favorable; noticing regression of symptoms and disappearance of pericardial effusion within two weeks. Clinical discussion: Acute pericarditis has been widely described in literature as probable complication of COVID-19, yet only few articles have reported it as a primary manifestation of COVID-19. Conclusion: Chest pain could be the only presenting symptom of COVID-19 among young, healthy individuals.To that end, clinicians should recognize cardiac involvement of COVID-19 and act accordingly to isolate patients and further limit the spread of the disease.

## Highlights

• The covid-19 is a global pandemic that is responsible for millions of deaths.
• Cardiovascular involvement in COVID-19 is less recognized and described.
• Acute pericarditis as the primary manifestation of COVID-19 is rare.
• Colchicine is the main treatment of pericarditis related to COVID-19.

## 1 Introduction

The early cases of coronavirus disease emerged back in Wuhan supermarket, China in November 2019. With its rapid transmission, the COVID-19 soon became a global pandemic leaving more than 2 million deaths around the world [1]. The typical clinical presentation of COVID-19 is characterized by respiratory tract symptoms that include fever, cough, fatigue or dyspnea. Cardiovascular involvement in COVID-19 is less recognized and described. This report aims to present the case of acute pericarditis as the primary manifestation of COVID-19, in the absence of respiratory symptoms among a young healthy patient with no comorbidities. Recognizing cardiac manifestations of COVID-19 is essential to isolate patients, and further limit the spread of the disease. To the best of our knowledge, it's the first case of acute pericarditis revealing COVID-19 among a young patient with no medical history.

Our case report was written according to CARE guidelines [2].

### 1.1 Case presentation

A 36 year old patient was admitted to the emergency department with pressure like chest pain. The pain was worse when lying on the back or when he took deep breath. To lessen his pain the patient leaned forward. He had also reported dyspnea at rest which occurred 3 days prior to his admission. The patient was a bodybuilder weighing 120 kg with no medical or surgical history nor drug history. In addition, there was no similar cases in the patient's family. On examination, the patient was conscious and hemodynamically stable. His heart rate was 80 b/m, blood pressure was 125/80 mm Hg. He was moderately polypneic with a respiratory rate of 26 breathts/min and an O2 saturation of 85–88% on ambient air. He had a fever measured at 38.8C. There were no signs of right cardiac failure. Further physical examination was normal.

**Fig. 1:**
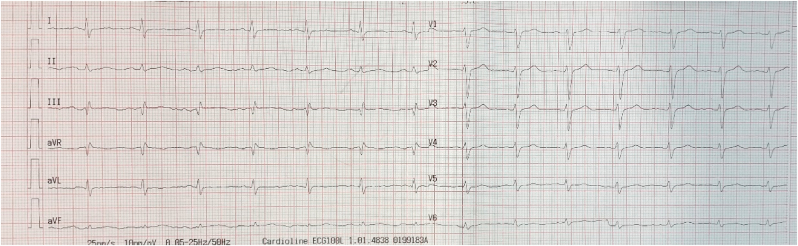
ECG showing no abnormalities.

**Fig. 2:**
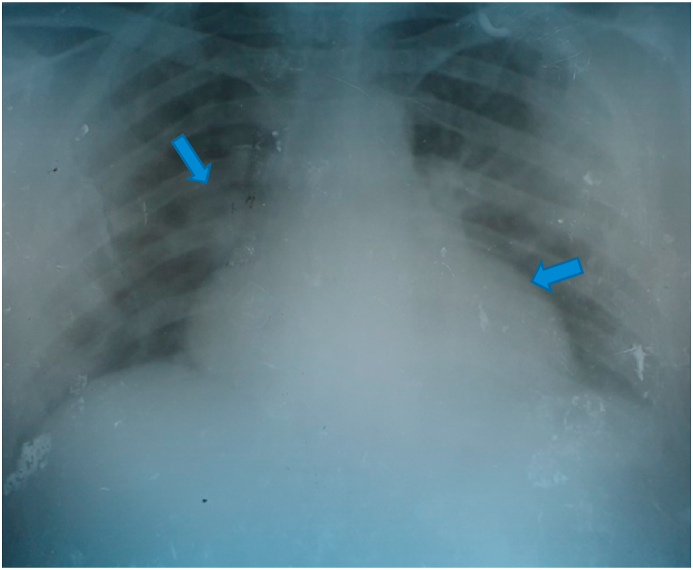
Chest xray revealing cardiomegaly and bilateral lung opacities.

**Fig. 3:**
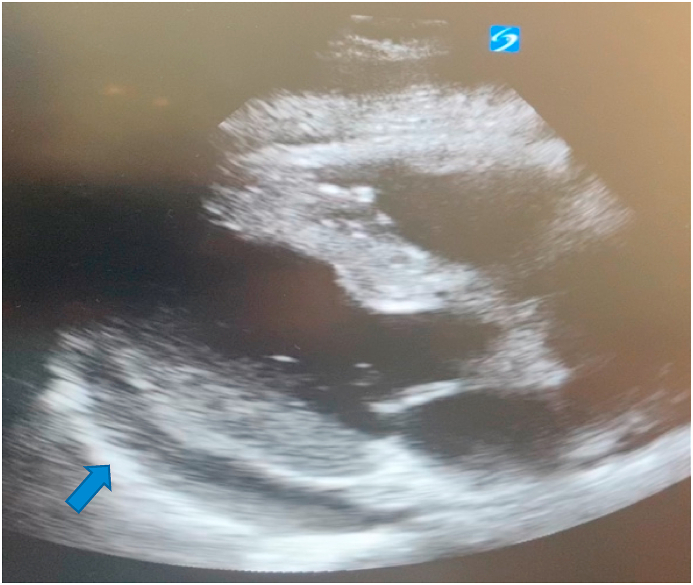
Parasternal long axis view, showing pericardial effusion.

**Fig. 4:**
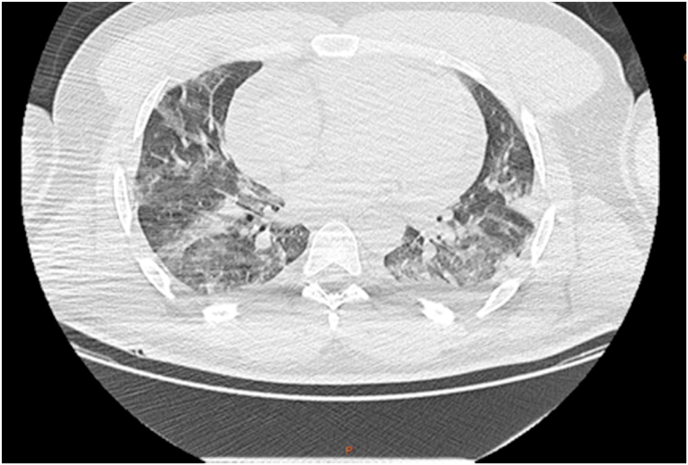
Crazy pavings and bilateral ground glass patterns.

The ECG showed no abnormality of cardiac muscle repolarization (PR and ST segments were normal) (Fig. 1). Chest X-ray however, revealed an enlarged cardiac index at 0.6 along with bilateral lung opacities (Fig. 2). Transthoracic echocardiogram (TTE) confirmed the presence of medium pericardial effusion at 12 mm in its largest measure (Fig. 3). It was decided to perform a thoracic CT scan based on the X-Ray findings and the epidemic context. The results showed pericardial effusion, bilateral ground glass pattern, in addition to crazy paving signs as it is most commonly seen in COVID patients. An estimated 50–75% involvement was noted (Fig. 4). Further serum and nasopharyngeal samples were sent for analysis which came back positive for COVID-19 infection.

Signs of inflammatory response were noted, white blood cells counted to 12,500/mm3 in addition to C reactive protein (CRP) at 120 mg/L, platelet counts were high up to 500,000 elements/mm3. Ferritin was elevated at 1800 μg/l. High sensitivity cardiac troponin T (hs-cTnT) on admission was within normal range. The kidney function was normal. Further investigations were realized to determine the origin of acute pericarditis, all came back negative (hepatitis B and C serology, EBV, Parvovirus 19, Epstein Barr, Cytomegalovirus, and Picornavirus).

Differential diagnoses included acute coronary syndrome, myocarditis and pneumonia.

The patient was admitted in the intensive care unit, where 10 L of oxygen were given using high concentration mask. The O2 saturation was 92%. The diagnosis of acute pericarditis was made based on clinical findings; typical chest pain and transthoracic echocardiogram. Colchicine was administered at 0.5 mg twice a day in addition of the conventional treatment of COVID-19 including the use of vitamin C 1g twice a day, vitamin D 25.000 UI per week, zinc 45mg twice a day, azithromycine 500 mg the first day then 250 mg per day. The tolerability was excellent and the patient did not report any adverse events during his hospitalization.

Within 4 days, chest pain disappeared. The O2 saturation was 92% using 4 L of nasal cannula. The patient was later on transferred to more stable COVID-19 unit, before being discharged at day 7. The transthoracic echocardiogram realized at day 15 showed no sign of pericardial effusion. The patient was very satisfied after the improvement in his clinical condition. Unfortunately after the first medical visit, he was lost to follow-up.

## 2 Discussion

Viral infections have been described to be the most common cause of myocarditis and pericarditis, however, few data is available to implicate the role of COVID-19 infection as the leading cause. In a recent article published in the *JAMA Cardiology*, authors have described myocarditis as possible cardiac complications in a 53-year-old woman with no respiratory tract symptoms [3], yet very few have reported the cases of pericarditis secondary to COVID-19 infection.

Acute pericarditis as the primary manifestation of COVID-19 has first been described by Kumar et al. in a 66 year old farmer with cardiovascular risk factors as hypertension, 40 pack-year smoking history and a significant familial premature coronary disease as opposed to our patient who was a healthy bodybuilder with no comorbidities or cardiovascular risk factors [4]. Chest pain is the main symptom that revealed pericarditis in both our case reports, and should therefore indicate possible cardiac involvement of COVID-19 infection. There is another case of a 47 year old COVID patient in the UK that presented as hemorrhagic pericardial effusion with tamponade, but he also had respiratory tract symptoms [5].

As it is well known, acute pericarditis is the inflammation of the pericardium that is frequently painful, and causes fluid to form and enter the pericardial space. Its causes are numerous, they include mainly viral infection in addition to cancer, anticoagulants, kidney failure and others [6]. Defining the underlying cause of pericarditis is not always easy. Blood tests and TTE are needed to help the physician assess an accurate diagnosis. Pericardiocentesis is the gold standard to determine the cause of pericardial effusion, but unfortunately not used on daily practices as it poses serious risk of cardiac injury.

Nonsteroidal anti-inflammatory drugs (NSAID) are the backbone therapy of acute pericarditis along with the use of colchicine. In cases of contraindication to NSAID, corticosteroids at low doses are used as second line option [7]. So far, no strong evidence has been reported regarding the use of NSAIDs and cardiovascular/respiratory adverse effects in COVID-19 patients. There is not sufficient data to advise against the use of NSAIDs if those are needed as it is the case in pericarditis. Furthermore, The European Medicines Agency (EMA) indicated that there is no scientific evidence supporting the use of ibuprofen to worsening condition of COVID-19 patients [8]. We chose however, to treat our patient using only colchicine at 0.5 mg twice a day, as it is not contraindicated for pericarditis in COVID-19 patients as it has been reported [4,8].

Notably in this case and in the absence of positive polymerase chain reaction to COVID-19 on pericardial fluid, the association between COVID-19 and an idiopathic pericarditis could not be excluded.

## 3 Conclusion

Cardiac involvement could be the sole and primary presentation of COVID-19 infection among young, healthy adults. Clinicians should be aware of this atypical manifestation, even in the absence of respiratory tract symptoms, and should act accordingly to isolate patients, push further investigations to limit the spread of COVID-19.

## Sources of funding

None.

## Ethical approval

The ethical committee approval was not required give the article type (case report).However, the written consent to publish the clinical data of the patients was given and is available to check by the handling editor if needed.

## Consent

Obtained.

## Author contribution

Raid Faraj: Study concept, Data collection, Data analysis, Writing the paper.

Chifaa Belkhayat: Study concept, Data analysis, Writing the paper.

Amine Bouchlarhem: Data collection.

Ghizlane El Aidouni: Contributor.

Houssam Bkiyar: Supervision and data validation.

Brahim Housni: Supervision and data validation.

## Research registration

This is not an original research project involving human participants in an interventional or an observational study but a case report. This registration is was not required.

## Guarantor

Raïd Faraj.

Chifaa Belkhayat.

## Provenance and peer review

Not commissioned, externally peer-reviewed.

## Declaration of competing interest

None.
